# Interaction-induced decay of a heteronuclear two-atom system

**DOI:** 10.1038/ncomms8803

**Published:** 2015-07-22

**Authors:** Peng Xu, Jiaheng Yang, Min Liu, Xiaodong He, Yong Zeng, Kunpeng Wang, Jin Wang, D. J. Papoular, G. V. Shlyapnikov, Mingsheng Zhan

**Affiliations:** 1 State Key Laboratory of Magnetic Resonance and Atomic and Molecular Physics, and Wuhan National Laboratory for Optoelectronics, Wuhan Institute of Physics and Mathematics, Chinese Academy of Sciences, Wuhan 430071, China; 2 Center for Cold Atom Physics, Chinese Academy of Sciences, Wuhan 430071, China; 3 School of Physics, University of Chinese Academy of Sciences, Beijing 100049, China; 4 INO-CNR BEC Center and Dipartimento di Fisica, Università di Trento, 38123 Povo, Italy; 5 Laboratoire de Physique Théorique et Modèles Statistiques, Université Paris Sud, CNRS, Orsay 91405, France; 6 Van der Waals-Zeeman Institute, University of Amsterdam, Science Park 904, 1098 XH Amsterdam, The Netherlands; 7 Russian Quantum Center, Novaya Street 100, Skolkovo, Moscow Region 143025, Russia

## Abstract

Two-atom systems in small traps are of fundamental interest for understanding the role of interactions in degenerate cold gases and for the creation of quantum gates in quantum information processing with single-atom traps. One of the key quantities is the inelastic relaxation (decay) time when one of the atoms or both are in a higher hyperfine state. Here we measure this quantity in a heteronuclear system of ^87^Rb and ^85^Rb in a micro optical trap and demonstrate experimentally and theoretically the presence of both fast and slow relaxation processes, depending on the choice of the initial hyperfine states. This experimental method allows us to single out a particular relaxation process thus provides an extremely clean platform for collisional physics studies. Our results have also implications for engineering of quantum states via controlled collisions and creation of two-qubit quantum gates.

## Abstract (web summary)

Understanding the behaviour of trapped two-atom systems is interesting for engineering quantum gases, and one of the key quantities to determine is the inelastic relaxation time from hyperfine states. Here, the authors measure this quantity for heteronuclear systems of ^87^Rb and ^85^Rb in a micro optical trap.

## Graphical abstract

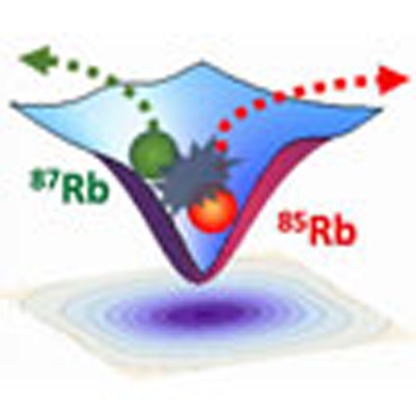

The studies of two-atom systems in small traps attract a great deal of interest, in particular for engineering of quantum states via controlled collisions and creation of quantum gates in quantum information processing with a set of single-atom traps^1^,^2^. The crucial points are the decoherence time and the lifetime related to the interaction-induced inelastic decay of a higher hyperfine state. On the other hand, this type of inelastic processes, in particular heteronuclear ones, are important for the creation of multi-species quantum degenerate systems^3^, for obtaining ultracold heteronuclear molecules^4^, and for ultracold chemistry^5^. In homonuclear systems, the inelastic processes have been well studied from large ensembles of atoms to a few and even two atoms^6^,^7^,^8^. However, in the studies of inelastic heteronuclear collisions in a trapped gas^9^ the main obstacle is the simultaneous presence of a large variety of loss mechanisms, which complicates the analysis. In magneto-optical traps where many heteronuclear systems have been studied^10^,^11^,^12^,^13^,^14^,^15^,^16^, aside from collisional processes one has radiative escape. In optical dipole traps there are homonuclear inelastic collisions^17^,^18^,^19^, and at sufficiently large densities, three-body recombination becomes important^20^. It is thus crucial to perform experiments allowing one to single out a particular inelastic process.

This is done in the present paper. We study a two-atom system of different isotopes of rubidium (single ^85^Rb and single ^87^Rb) in a micro optical trap. One of them or both are in a higher hyperfine state, and we measure the corresponding rate of inelastic relaxation accompanied by the loss of the atoms. The homonuclear collisions are absent and our measurements give pure loss rates of specific hyperfine heteronuclear collisions. The experiments are done at temperatures close to the border of the ultracold limit (tens of microkelvins) and are supported by finite temperature coupled channel calculations. Our work can be easily extended to other alkali atoms, even to atom–molecule collisions^21^,^22^, thus allowing further understanding of heteronuclear collisions, a precise test of atomic collisional theory, and applications to quantum information processing.

## Results

### Experimental setup and results

**Figure 1:**
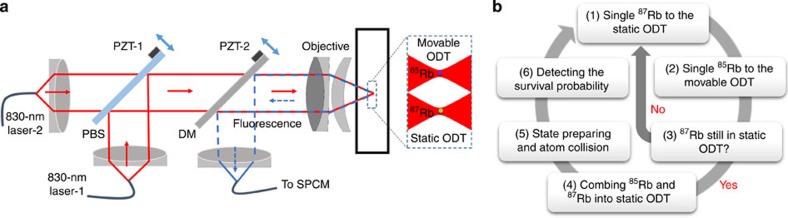
Experimental setup and measurement time sequence. (**a**) Schematic diagram of the experimental setup. Two 830-nm lasers are collimated, combined by a polarizing beam splitter (PBS) and then strongly focused by an objective (Linos, HALO30) into the vacuum chamber to form two ODTs. The movable ODT is from 830-nm laser-1 and can be shifted to overlap with the static ODT (from 830-nm laser-2) by controlling piezoelectric ceramic transducer (PZT)-1. The fluorescence of trapped single atoms is collected by the same objective, separated from dipole lasers by a dichroic mirror (DM) and guided to single-photon-counting module (SPCM) for detection. PZT-2 controls the fluorescence-collecting region. A detailed description can be found in Methods. (**b**) Time sequence in the experiment. Each survival probability in our experiment is the result from 300 repeated measurements.

Our two-atom heteronuclear system is composed of a single ^85^Rb and a single ^87^Rb in a micro optical dipole trap (ODT), and there are three important points in the experiment. The first one is a sequential trapping of a single ^87^Rb in a static ODT and a single ^85^Rb in a movable ODT^23^, and we make sure that two atoms of different isotopes are actually trapped (see Fig. 1 and Methods). Second, we shift the movable ODT to overlap with the static one, and adiabatically turn off the movable trap. We get ^87^Rb and ^85^Rb in one trap with probability of about 95%. The third point is that the collisional blockade^24^ does not allow us to detect the presence of ^87^Rb or ^85^Rb when both of them are in the same trap. Therefore, we first have to kick out one of the atoms to detect the presence of the other one. By optimizing this procedure we have minimized unwanted atom losses to <3%.

Depending on the hyperfine states of ^87^Rb and ^85^Rb, there are three inelastic decay processes:


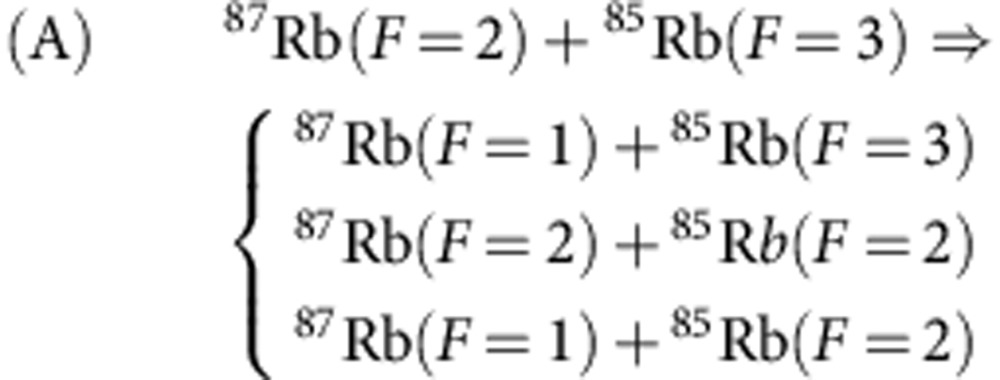



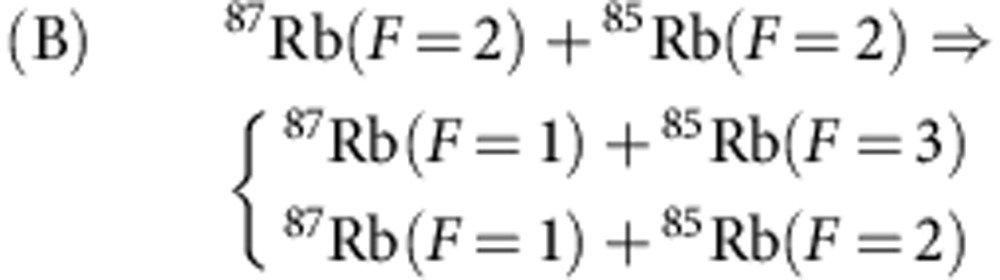



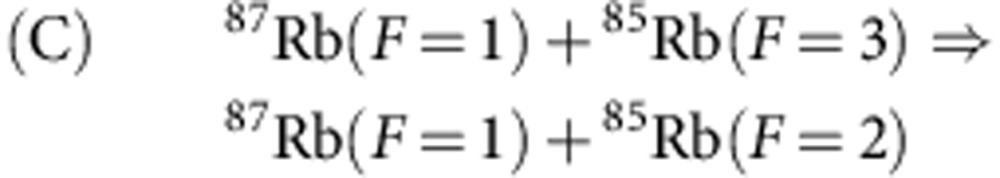


We have not set a magnetic field, and for each atomic spin *F* in the initial state of the collision the states with all possible values of the spin projection *M*_*F*_ are likely equally populated. The energy released in the inelastic processes A, B and C is about several GHz and it exceeds the trap depth *U*_0_ by more than two orders of magnitude. Therefore, both atoms are ejected from the trap as a result of the inelastic relaxation. In most of our experiments the trap depth is *U*_0_=0.6 mK, which in our configuration provides the radial trap frequency *ω*_*ρ*_/(2*π*)=38.8±0.1 kHz and the axial trap frequency *ω*_*z*_/(2*π*)=3.2±0.1 kHz.

**Figure 2:**
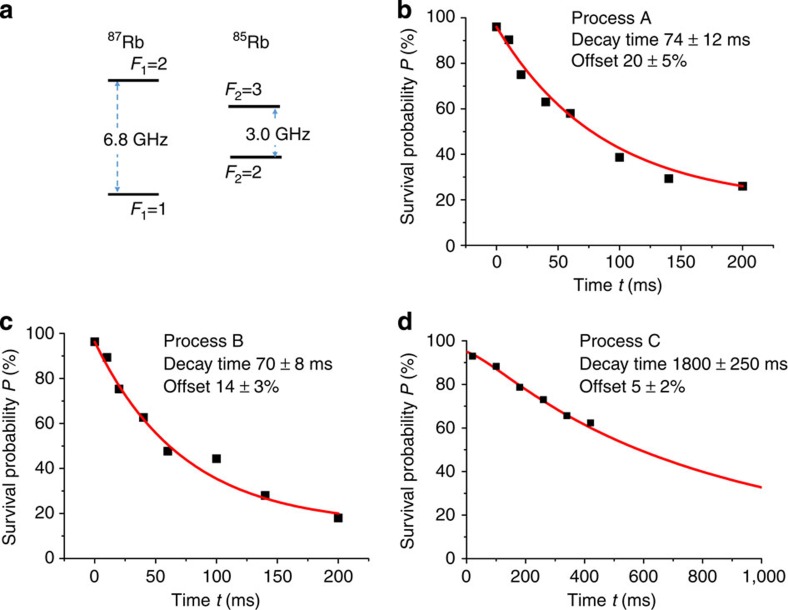
Experimental data for the decay rates. (**a**) Energy levels of hyperfine states of ^87^Rb and ^85^Rb. (**b**–**d**) Survival probability *P* versus time *t* for the A, B and C collisions, respectively. The measurements are done for the survival probability of ^87^Rb after kicking out ^85^Rb. The black squares are experimental data, with each point being the result from 300 repeated measurements. In (**b**,**c**) the solid curves show a fit by the formula *P*=*w* exp(−*t*/*τ*)+*w*_0_, and the error in the decay time indicates the s.d. when using the fit of *P*(*t*) by the exponential formula. In (**d**) the solid curve is a fit with the numerical solution of the rate equations including single-atom spin relaxation. The error in the decay time shows the uncertainty originating from the uncertainty in the single atom spin relaxation time *τ*_r_ entering the rate equations (see Methods). The data are collected at the trap depth *U*_0_=0.6 mK and the initial temperatures *T*_87_=35±3 μK, *T*_85_=15±1 μK.

We measure the survival probability *P*(*t*) for the atoms to remain in the trap at time *t* (see Fig. 2). For each *t* we execute 300 repetitions of the loop sequence of Fig. 1b. In the case of A and B processes the decay is strongly dominated by the interaction-induced spin relaxation. The probability *P*(*t*) is then described by an exponential time dependence. Within <10% of uncertainty the experimental data can be fitted with an exponential function *P*=*w* exp(−*t*/*τ*)+*w*_0_. The presence of the offset *w*_0_ has several reasons discussed in detail in Methods. First of all, the two-atom system is obtained with 95% probability, and there are traps with only one atom that remains trapped on a much longer timescale (about 11 s (ref. 25)) than the collisional lifetime *τ*. Second, for the A and C processes doubly polarized pairs (for each atom the spin projection is equal to the spin) can decay only due to weak spin–spin or spin–orbit interactions that may change the spin projection of the pair, and the polarized pairs practically remain stable on the timescale of our experiment. For the B process, however, the doubly polarized pairs efficiently relax due to the channel leading to the formation of ^87^Rb(*F*=1) and ^85^Rb(*F*=3).

The process C is much slower than A and B, and our measurements for this process have been made on a timescale of about 500 ms. In this case the decay is significantly influenced by single-atom spin relaxation, and we have to take it into account in the rate equations for extracting *τ* from our measurements (see Methods).

The measured *τ* has about 15% of statistical uncertainty that decreases with increasing the executed loop numbers. Aside from single-atom spin relaxation, the value of *τ* is influenced by single-atom loss events. The heating rate in the dipole trap is about 20 μK s^−1^ (ref. 26) and it increases the collisional volume, thus slightly increasing the decay time *τ*. We estimate the overall uncertainty in our values of *τ* as about 20%.

**Figure 3:**
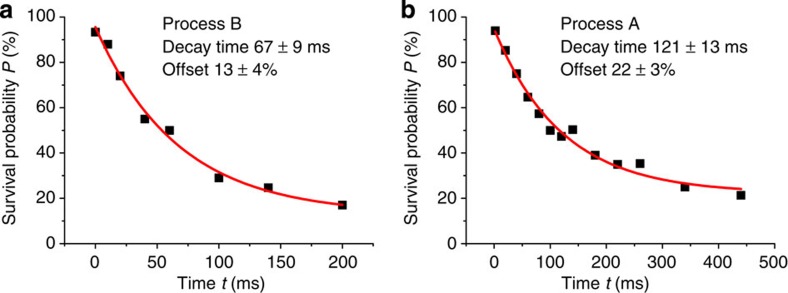
The decay under different conditions. (**a**,**b**) Survival probability *P* versus time *t* for the B and A collisions, respectively. The black squares are experimental data collected at the trap depth *U*_0_=0.6 mK, with each point being the result from 300 repeated measurements. The solid curves show a fit by the formula *P*=*w* exp(−*t*/*τ*)+*w*_0_, and the error in the decay time indicates the s.d. when using the fit of *P*(*t*) by the exponential formula. In (**a**) The measurements are done for the survival probability of ^85^Rb after kicking out ^87^Rb, and the initial temperatures are *T*_87_=35±3 μK and *T*_85_=15±1 μK. In (**b**) ^85^Rb is kicked out, and the survival probability of ^87^Rb is measured with the initial temperatures *T*_87_=47±3 μK and *T*_85_=27±2 μK.

We also test that the result for *τ* does not depend on whether we kick out ^85^Rb or ^87^Rb for measuring *P*(*t*). Comparing Fig. 2c with Fig. 3a it is easy to conclude that not only relaxation times are very close to each other (see also Table 1) but also the functions *P*(*t*). We then vary the temperature for the A collisional process to test the dependence of *τ* on the effective volume (density) of atoms in the trap. As expected, the time *τ* increases with temperature and one can see this from the comparison of the results in Figs 2b and 3b.

### Theory and analysis

The rate equations for the inelastic decay processes A, B and C can be written as


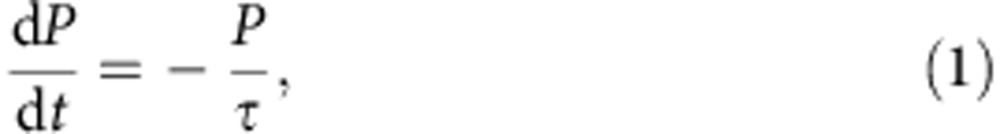


where *P*(*t*) is the probability that at time *t* the atoms are still present in the trap, and *τ* is the relaxation (decay) time that we measure. These processes occur at interatomic distances of the order of or smaller than the radius of the interaction potential *R*_e_=(*mC*_6_/*ħ*^2^)^1/4^≈80 Å (*C*_6_ is the Van der Waals constant). At our trap frequencies and temperatures from 15 to 55 μK we have 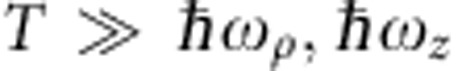, and the motion of atoms in the trap is surely quasiclassical. In our trap configuration the extension of the wavefunction of the trapped atoms is the shortest in the radial direction 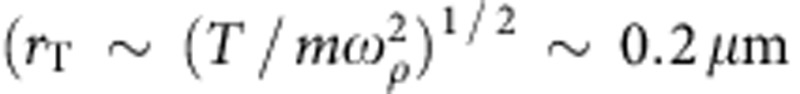, where *m* and *ω*_*ρ*_ are the atom mass and radial trap frequency, respectively), and we still have the inequality


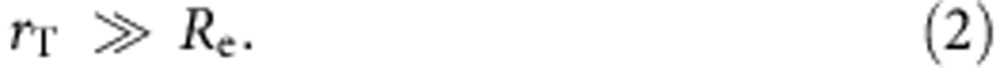


Therefore, the decay (relaxation) time *τ* can be expressed through the relaxation rate constant *α* in free space:


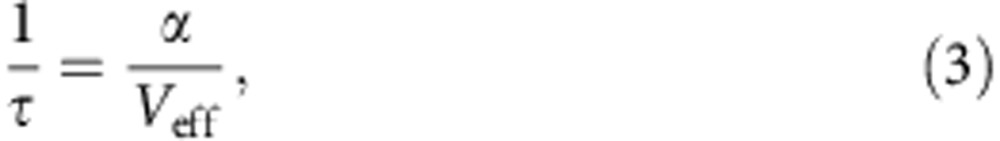


where


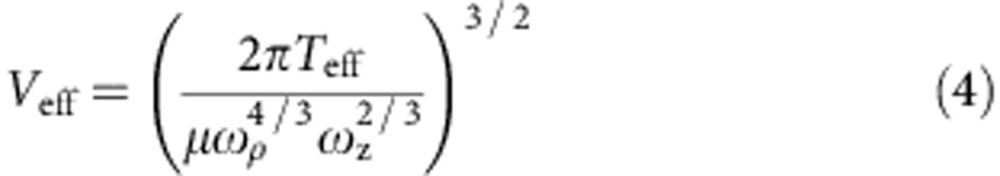


is the effective volume, *T*_eff_=*μ*(*T*_1_/*m*_1_+*T*_2_/*m*_2_) is the effective temperature, with *m*_1_, *m*_2_ and *T*_1_, *T*_2_ being the masses and temperatures of ^87^Rb and ^85^Rb, respectively, and *μ* is the reduced mass. The rate constant *α* in equation (3) is averaged over the Boltzmann distribution of relative momenta *k* at effective temperature *T*_eff_:


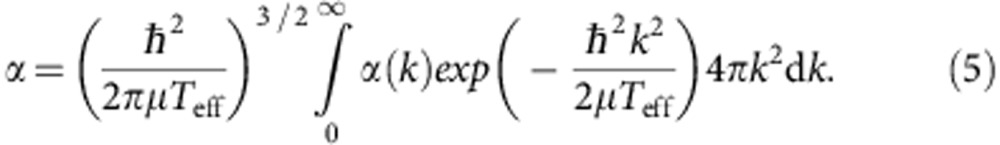


Note that due to elastic collisions between the atoms, the two-atom system eventually acquires an equilibrium temperature (*T*_1_+*T*_2_)/2. However, it is different from the initial effective temperature *T*_eff_ by <1%, and so will be the effective volume and the average value of *α*.

At our temperatures, the quantity *k*_T_*R*_e_∼0.5, where *k*_T_=(*mT*/*ħ*^2^)^1/2^ is the thermal momentum, so that we are close to the border of the *s*-wave-scattering limit. Therefore, in addition to the *s*-wave scattering, we took into account the scattering with higher orbital angular momenta. The rate constants *α*_A_, *α*_B_ and *α*_C_ for the processes A, B and C were calculated using the coupled channel method^4^,^27^ at finite collision energies (see Methods). In the centre of mass reference frame, the Hamiltonian governing the collisions has the form


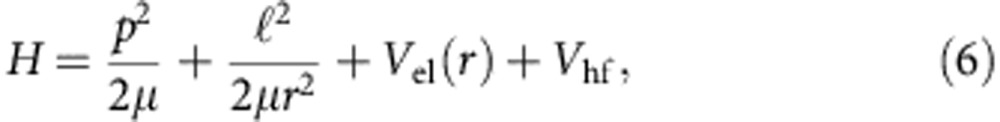


where *r* is the interatomic distance, *p* is its conjugate momentum and *ℓ* is the orbital angular momentum operator. The interatomic interaction operator is given by *V*_el_(*r*)=*V*_s_(*r*)*P*_s_+*V*_t_(*r*)*P*_t_, with *P*_s_ and *P*_t_ being projectors onto the electronic singlet and triplet states of the colliding pair of atoms. The term *V*_hf_=*a*_1_**S**_1_·**I**_1_+*a*_2_**S**_2_·**I**_2_ is the hyperfine interaction, where **S**_1_, **I**_1_, *a*_1_ and **S**_2_, **I**_2_, *a*_2_ are the electron and nuclear spin operators and hyperfine constants for ^87^Rb and ^85^Rb, respectively. The total spin operators of the atoms are **F**_1_=**S**_1_+**I**_1_ and **F**_2_=**S**_2_+**I**_2_, and the total spin operator of the pair is **F**=**F**_1_+**F**_2_. The Hamiltonian *H* of equation (6) conserves both the total spin *F* and its projection *M*_*F*_. It also conserves the orbital angular momentum *ℓ* and its projection *M*_*ℓ*_.

The rate of inelastic spin relaxation occurring when at least one of the colliding atoms is in a higher hyperfine state can be expressed through the real and imaginary parts of the elastic scattering amplitude. The well-known formula for the inelastic rate constant^28^, averaged over the initial spin projections, is transformed to (see Methods):


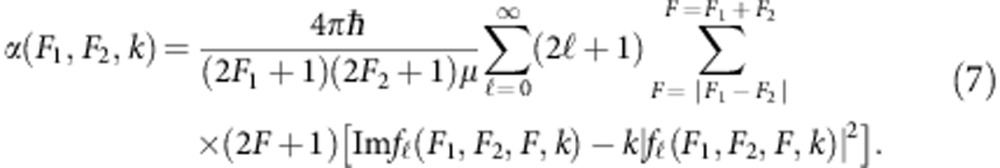


Accordingly, *α*_A_≡*α*(2, 3), *α*_B_≡*α*(2, 2), and *α*_C_≡*α*(1, 3). The quantity *f*_*ℓ*_(*F*_1_, *F*_2_, *F*, *k*) in equation (7) is the amplitude of elastic *ℓ*-wave scattering of these atoms at the total spin *F*.

We apply the accumulated-phase method (see Methods) and calculate the accumulated-phase parameters from the known properties of homonuclear ^87^Rb^87^Rb and ^85^Rb^85^Rb collisions using mass scaling^27^. The main inaccuracy of our calculations stems from the choice of the accumulated phase, and we have checked that our results are stable within 5% when the value of this phase is varied by a few percent. The *p*-wave (*ℓ*=1) contribution at our temperatures is comparable to the *s*-wave (*ℓ*=0) one, but the contributions of the *d*-wave and higher partial waves are below 1%. Therefore, in the following we confine ourselves only to the *s*-wave and *p*-wave scattering.

In Table 1 the results of the calculations for the processes A, B and C are compared with the experimental data. To show that finite temperature effects are important in our experiment we also present the rate constants calculated at *T*=0. For the fast A and B processes one sees an agreement between experiment and theory within the error bars of the experimental data. For the slow C process the calculated *α* is near the upper bound of the experimental value accounting only for statistical uncertainties. The reason for this small discrepancy is that the heating effect, although fairly small, still increases the effective volume so that the measured *τ* actually corresponds to slightly higher temperatures than the initial ones. This means that the experimental value of *α* at the initial temperatures should actually be slightly (by about 15%) higher than the one in Table 1.

## Discussion

The relaxation rates obtained in our work are rather high. In interesting experiments with spinor heteronuclear mixtures^29^, this places an upper limit of about *n*∼10^12^ cm^−3^ on the density if a higher hyperfine atomic state is involved. We should note, however, that the rate constant *α*_C_ has a pronounced temperature dependence. At the temperatures used in this experiment, it is larger by about a factor of 4 than at *T*=0 (see Table 1). Our zero temperature result *α*_C_=0.8 × 10^−12^ cm^3^ s^−1^ is somewhat below the lower bound of the interval (1.2–4.5) × 10^−12^ cm^3^ s^−1^ found for the C process with ^87^Rb(*F*_1_=1, *M*_1_=−1) and ^85^Rb(*F*_2_=3, *M*_2_=3) in ref. 30, which used old data for the interaction potentials. In experiments with ultracold clouds containing many atoms, the common temperature is ∼100 nK, which practically corresponds to *T*=0 limit in our calculations. We thus expect that an admixture of ^85^Rb(*F*_2_=3) can be collisionally stable on a timescale of 100 ms in the gas of ^87^Rb(*F*_1_=1) at densities approaching 10^13^ cm^−3^.

Two-atom systems in small traps are convenient for the creation of heteronuclear molecules. In the gas mixture, Feshbach molecules of ^87^Rb(*F*_1_=1, *M*_1_=−1) and ^85^Rb(*F*_2_=2, *M*_2_=−2) have already been created^31^ and they were undergoing losses due to collisional relaxation. In the two-atom system in a trap, the Feshbach molecule can be created by sweeping the magnetic field across the resonance like in experiments in an optical lattice^32^. For ^87^Rb–^85^Rb, Feshbach resonances have been observed in the fields of hundreds of Gauss^31^ and also predicted in the fields below 10 G ref. 33. The rate of a spontaneous decay (dissociation) of a single Feshbach molecule in a trap due to spin relaxation^34^,^35^ is proportional to *α*/〈*r*〉^3^, where *α* is the rate constant of relaxation in binary collisions and 〈*r*〉 is the size of the molecule^35^. With our relaxation rate constants *α*∼10^−10^ to 10^−12^ cm^3^ s^−1^ the decay time can be made on the level of tens of milliseconds, like in the case of ^85^Rb–^85^Rb Feshbach molecules^35^. This is sufficient for using the two-photon-stimulated Raman adiabatic passage and transferring this molecule to the ground rovibrational state^36^.

Our results may also have implications for engineering of quantum states and creation of two-qubit quantum gates based on controlled collisions in a system of single-atom traps^1^,^2^,^37^, in particular with respect to a proper selection of hyperfine atomic states. In our system, the relaxation time is *τ*=*V*_eff_/*α* and it strongly decreases with temperature, so that for atoms in the ground vibrational state *τ* may become of the order of the single-atom decoherence time *τ*_dc_. For collisional quantum gates the time *τ*_dc_ and, hence, *τ* should be at least four orders of magnitude larger than the operational time *τ*_op_ (ref. 38). The latter cannot be much smaller than the inverse interaction energy of the two atoms in the trap, and for atoms in the ground vibrational state one has *τ*_op_∼*ħV*_eff_/*g*, where *g*=4*πħ*^2^*a*/*m* is the coupling constant for the elastic interaction, and *a* is the scattering length (this leads to *τ*_op_ of about tens of microseconds at our trap frequencies, although for our present temperatures it is on the millisecond level). We thus have the condition:


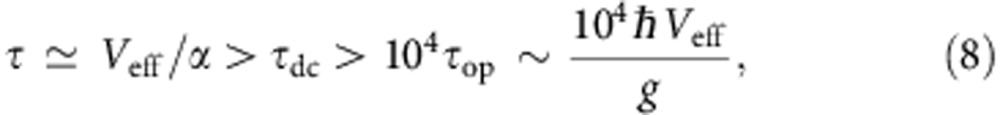


which immediately leads to the inequality


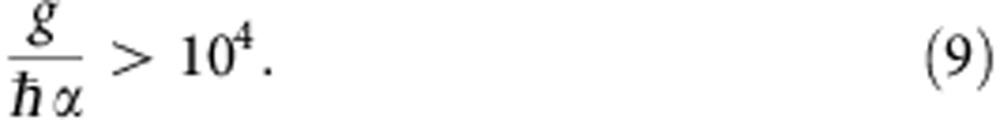


For common values of *g*/*ħ* from 10^−9^ to 10^−11^ cm^3^ s^−1^ equation (9) can be satisfied for doubly polarized atomic states, such as ^87^Rb(*F*=2, *M*_*F*_=2) where *g*/*ħ*∼10^−10^ cm^3^ s^−1^, and the relaxation is caused only by weak spin–spin or spin–orbit interactions^32^ leading to *α*<10^−14^ cm^3^ s^−1^ Another option would be to increase *g* by using a Feshbach resonance, although this can also increase inelastic losses.

The advantage of our work is that we study collisions in a two-atom system, which allows us to single out a particular collisional process. In upcoming experiments we intend to trap single atoms with a farer detuned dipole laser (1,064 nm) to suppress the single-atom spin relaxation. We then prepare atomic states with given spin projections *M*_*F*_ and cool the atoms down to the ground vibrational state^39^,^40^ to execute the possibility of creating a quantum gate. Our work also paves a way to the creation of single heteronuclear molecules and to the studies of atom–molecule and molecule–molecule binary systems.

## Methods

### Details of experimental methods

In our experiment single atoms are trapped in micro ODTs that are formed by strongly focusing 830-nm lasers to a beam waist of about 2.1 μm. The dipole lasers follow the paths shown in Fig. 1a by the red solid lines. The movable ODT is initially 5 μm away and it can be shifted to overlap with the static ODT by changing the voltage of piezoelectric ceramic transducer-1. We detect trapped atoms by collecting the fluorescence in the trap region as shown by blue dashed lines. The fluorescence is coupled into a polarization-maintaining single-mode fibre (with the core diameter of 5 μm) for spatial filtering and is then guided to a single-photon-counting module (AQRH-14-FC). Owing to a moderate core diameter of the fibre and to a fairly large distance between the two traps, we can selectively collect the fluorescence from one of them. The disturbance from the other trap (crosstalk effect) is eliminated by properly adjusting the voltage of piezoelectric ceramic transducer-2.

The experiment is executed following the time sequence shown in Fig. 1b. We first trap ^87^Rb in the static ODT. During this step the movable ODT is off to prevent the loading of ^87^Rb. Then we turn off the magneto-optical trap (MOT) of ^87^Rb, and turn on the movable ODT and the ^85^Rb MOT to execute step 2. The loading of a single ^85^Rb may occur not only into the movable ODT but also into the static ODT, which leads to the loss of both ^85^Rb and ^87^Rb due to collisional blockade. Therefore, we do an extra check of the ^87^Rb presence in the static trap in step 3. Only when ^87^Rb is still trapped, we record the final result of the collision, otherwise we go back to step 1 and start loading again. To check that only ^87^Rb is in the static trap and meanwhile only ^85^Rb in the movable trap, we first detect the absence of the fluorescence of ^87^Rb from the movable trap and the absence of fluorescence of ^85^Rb from the static trap. This is followed by the fluorescence detection of ^87^Rb and ^85^Rb in the static and movable traps, respectively.

In step 4 we first prepare ^85^Rb in *F*_2_=2 and ^87^Rb in *F*_1_=1 states to eliminate the unwanted collisional losses when switching off the MOT repumping lasers 1 ms before the MOT cooling lasers. For minimizing the heating effect we have optimized the process of transferring ^85^Rb to the static trap. The transfer efficiency can go up to 98% and is limited by the detection efficiency and heating losses. The probability that ^87^Rb survives when the movable trap approaches the static one and then is adiabatically switched off is also about 98%. The temperature is measured in this type of process by using the release and recapture technique^41^,^42^ when one of the traps (either static or movable) is empty.

In this way we create a heteronuclear two-atom system. After a certain time, we kick out one of the atoms from the trap using resonant lasers. Optimizing the laser intensities and shortening the pulse duration to 0.1 ms, we have minimized the unwanted losses to <3%. Eventually, we have succeeded in trapping two heteronuclear single atoms in the static ODT with about 95% probability.

### Analysis of the experimental data

To confirm that the decay times *τ*_A_ and *τ*_B_ can be extracted by a simple exponential fit of the experiment data in Figs 2b,c and 3a,b, we numerically solved the rate equations taking into account single-atom spin relaxation. These are linear equations for the quantities 
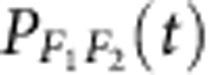
 representing the probabilities that both ^87^Rb with spin *F*_1_ and ^85^Rb with spin *F*_2_ are present in the trap at time *t*:


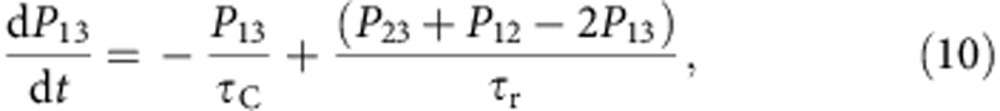



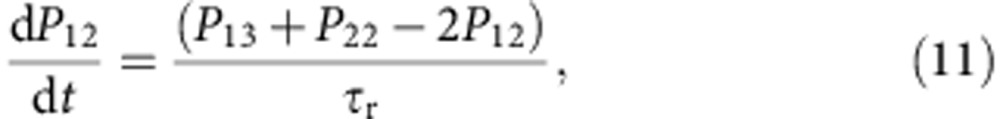



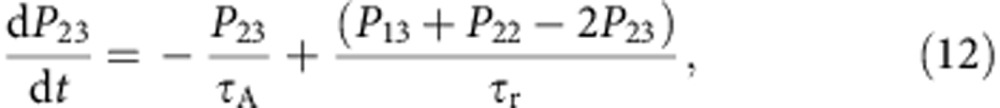



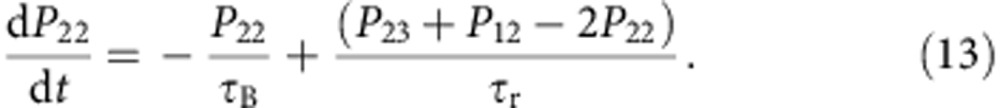


**Figure 4:**
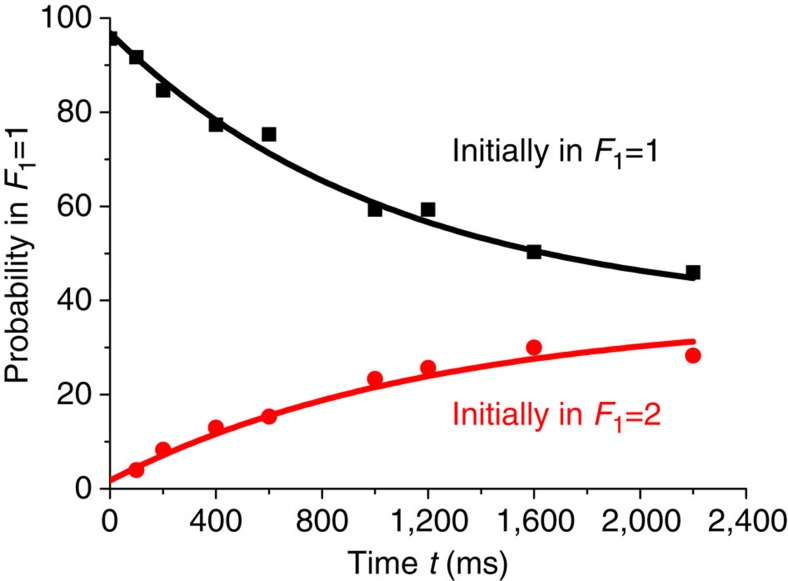
Spin-relaxation measurement. We first prepare a single ^87^Rb atom in the state *F*_1_=1 (black squares) or *F*_1_=2 (red circles). After a time *t* we kick out atoms that are populated in the state *F*_1_=2 by using a resonant laser and then detect the survival probability of single atoms. Each data is averaged over 300 single atoms. The solid curves show a fit by the exponential formula: d*P*/d*t*∝exp(∓*t*/*τ*_r_ −*t*/*τ*_s_), where the sign − is related to the black curve, the sign + to the red one, and the single-atom loss time is *τ*_s_=11,000 ms. The fitted spin-relaxation time is *τ*_r_=1,100±150 ms.

We do not include single-atom loss in the rate equations, as the related loss time is about 11 s and it changes the simulated survival probability by <2% even at times *t* approaching 1,000 ms. For the time of single-atom spin relaxation we did an independent measurement, and the measured value is *τ*_r_=1,100±150 ms (see Fig. 4).

The best fit with the experimental data for the process A in the conditions of Fig. 3b is obtained with *τ*_A_=129.5±1.5 ms at *τ*_B_=115±15 ms, *τ*_C_=1,800 ms and *τ*_r_=1,100 ms. The obtained *τ*_A_ is consistent with the decay time of 121±13 ms given in Fig. 3b from the simple exponential fit. The simulations in the conditions of Figs 2b,c and 3a show that the best fitted *τ*_A_ and *τ*_B_ are larger than the exponential-fit decay times indicated in these figures by not more than 2 ms.

The offsets of the fitted decay curves for the A and B processes in Figs 2 and 3 originate from several effects. They include the probability that there is only one atom in the trap ((5±2)%), the probability that ^87^Rb(*F*_1_=2) undergoes a transition to the state with *F*_1_=1 due to Raman scattering of the dipole laser (about 5% for Figs 2b,c and 3a, and about 7% for Fig. 3b), and for the A process the 6% probability that atom pairs are prepared in a doubly polarized state. All the offsets can be explained by these effects within our measurement uncertainty.

**Figure 5:**
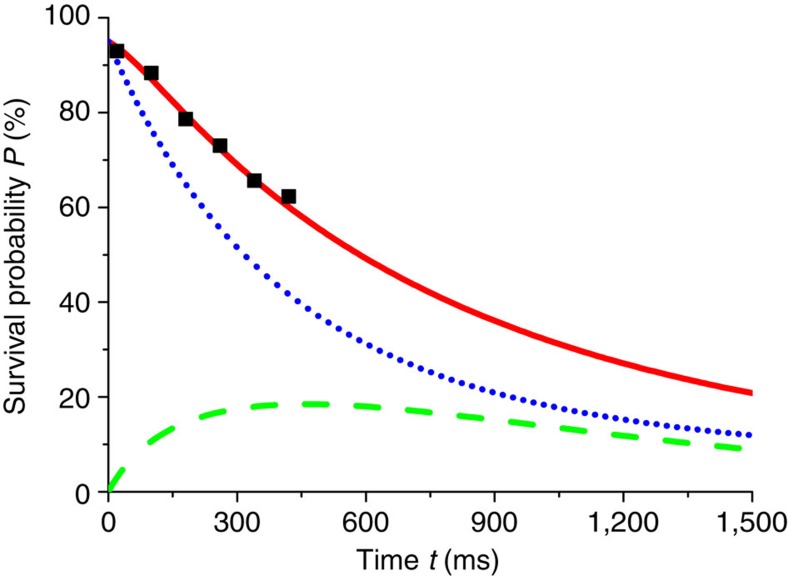
Numerical fitting for the process C. We fit the experimental data (black squares) by numerical results from the rate equations for the process C. The red solid curve shows the survival probability of ^87^Rb, the blue dotted curve shows the probability *P*_13_, and the green dashed curve is the probability *P*_23_+*P*_22_+*P*_12_.

For the slow process C the timescale is longer and the single-atom spin relaxation is more important, so that the time *τ*_C_ can be extracted only from the best fit of the numerical solution of the rate equations with the experimental data. In Fig. 5 we display the results of the simulations in the conditions of Fig. 2d up to *t*=1,500 ms. The best numerical fit deviates from the experimental data by <10%.

### Calculation of the inelastic rate constants

At low temperature the inelastic spin relaxation occurs when at least one of the colliding atoms is in a higher hyperfine state. The rate constant of this process is given by^28^


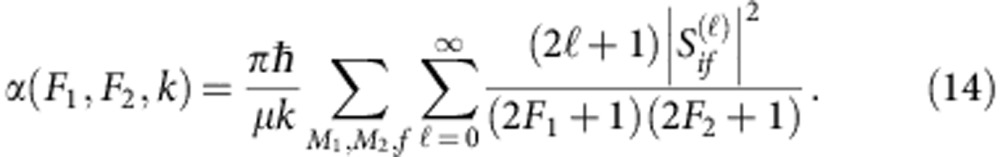


The quantity 
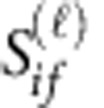
 is the *S*-matrix element for the *ℓ*-wave scattering from the initial state *i* characterized by the atom spins *F*_1_, *F*_2_ and their projections *M*_1_, *M*_2_ to a final state *f* that has a lower internal (hyperfine) energy, so that there is an energy release in the inelastic scattering process. In equation (14) we also averaged over the initial spin projections *M*_1_ and *M*_2_. Owing to the unitarity condition for the *S*-matrix elements we have:


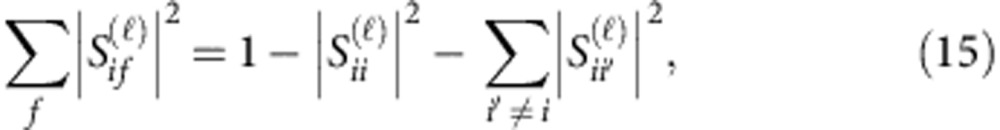


where *S*_*ii*_ is the *S*-matrix element for elastic scattering in which the spin projections *M*_1_, *M*_2_ remain the same, and *S*_*ii*′_ is the *S*-matrix element for elastic scattering, which changes *M*_1_, *M*_2_ to 
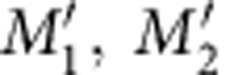
. Expressions for the *S*-matrix elements through the corresponding scattering amplitudes read^28^:


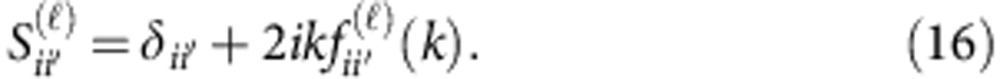


The amplitudes 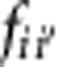 are conveniently expressed through the amplitudes *f*_*ℓ*_(*F*_1_, *F*_2_, *F*, *k*) of elastic scattering of atoms with spins *F*_1_, *F*_2_ at the total spin *F* of the pair:


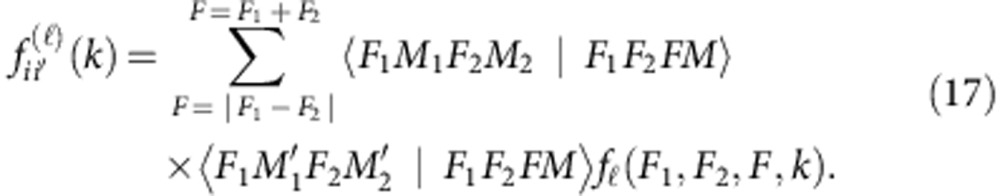


In the absence of a magnetic field the amplitudes *f*_*ℓ*_(*F*_1_, *F*_2_, *F*, *k*) do not depend on the total spin projection *M*. The Clebsch–Gordan coefficients, which appear in equation (17), satisfy the summation rule:


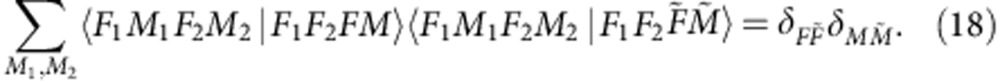


Substituting equations (16) and (17) into equation (14), and making use of equation (18), we arrive at equation (7) of the main text:


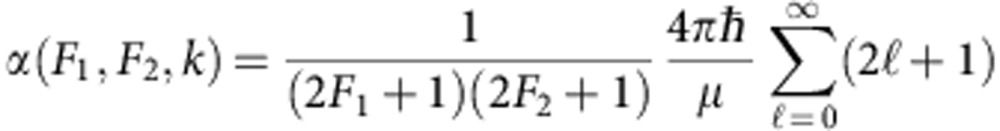



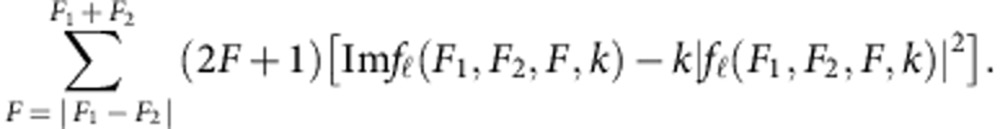


The amplitudes *f*_*ℓ*_(*F*_1_, *F*_2_, *F*, *k*) were calculated numerically using the coupled channel method^4^. Our implementation of this method is described in ref. 43. The asymptotic behaviour of the scattering states is enforced at a distance of 1,000*a*_0_, with *a*_0_ being the Bohr radius. The accumulated-phase boundary condition is applied at *r*_0_=16*a*_0_. It summarizes the short-range physics in the region of distances *r*<*r*_0_, where the triplet and singlet interaction potentials are poorly known, into six phase parameters. We calculate these parameters for heteronuclear ^87^Rb^85^Rb collisions starting from the known data for homonuclear ^87^Rb^87^Rb and ^85^Rb^85^Rb collisions and using the mass scaling technique^27^, which exploits the fact that the Born–Oppenheimer electronic potentials *V*_*s*_ and *V*_*t*_ do not depend on the type of isotopes. The hyperfine coefficients *a*_1_ and *a*_2_ were taken from ref. 44. Solving the coupled differential equations for the wavefunctions within the subspaces characterized by the conserved quantum numbers *F*, *M* we calculated all scattering amplitudes *f*_*ℓ*_(*F*_1_, *F*_2_, *F*, *k*) as functions of the incident relative momentum *k*. The calculated rate constants *α*(*F*_1_, *F*_2_, *k*) are then averaged over the thermal distribution of *k* according to equation (5).

**Figure 6:**
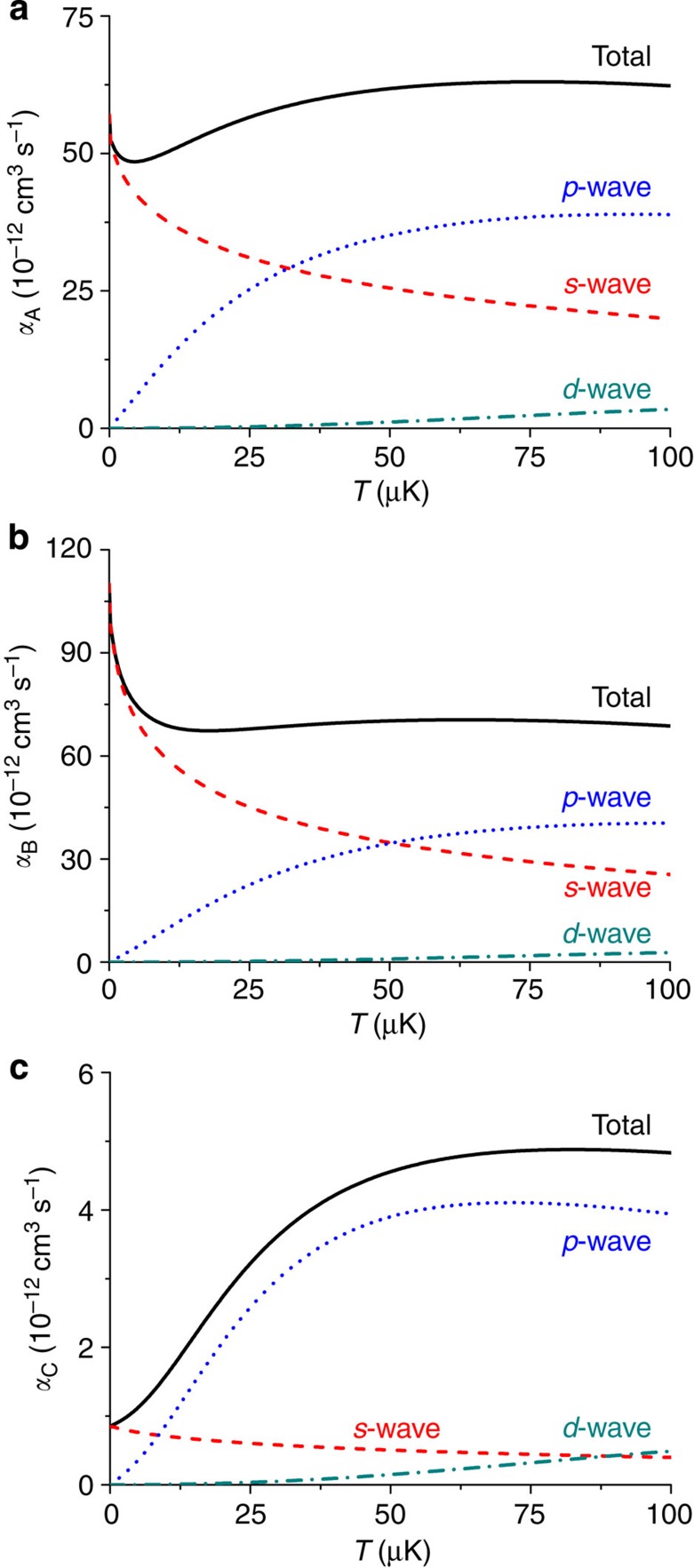
Calculated rate constants. (**a–c**) The rate constants *α*_A_, *α*_B_ and *α*_C_ are calculated at temperatures ranging from 0 to 100 μK, respectively. The black solid curves show the total rate constant, and the red dashed, blue dotted and dark cyan dash–dotted curves show the *s*-wave, *p*-wave and *d*-wave contributions, respectively.

In Fig. 6 we present the calculated rate constants at temperatures from 0 to 100 μK and specify the *s*-wave, *p*-wave and *d*-wave contributions (the latter is below 1% at temperatures of our experiment, but at *T*=100 μK it is 4–5% for the A and B processes, and 10% for the C process). As one can see, the *s*-wave contribution slowly decreases with increasing temperature. However, the *p*-wave contribution significantly grows with *T*, which is expected. The role of the *p*-wave scattering is especially important for the C process, where it starts to dominate over the *s*-wave scattering already at *T*∼15 μK. As a result, the total *α*_C_ significantly increases with temperature. For the A and B processes the total *α* does not change much with increasing *T* as the decrease in the *s*-wave contribution is compensated by the *p*-wave contribution.

## Additional information

**How to cite this article:** Xu, P. *et al.* Interaction-induced decay of a heteronuclear two-atom system. *Nat. Commun.* 6:7803 doi: 10.1038/ncomms8803 (2015).

## Figures and Tables

**Table 1:** Summary of the experimental and calculated results. The experiment is done with the trap depth *U*_0_=0.6 mK (*ω*_*ρ*_/(2*π*)=38.8 kHz, *ω*_z_/(2*π*)=3.2 kHz).

**Collisional process**	**Experimental parameters: temperature (μK)**	**Decay time (ms)**	**Experimental** ***α*****(cm**^**3**^ **s**^**−1**^**)**	**Calculated** ***α*****(cm**^**3**^ **s**^**−1**^**)**	**Calculated** ***α***_***T*****=0**_**(cm**^**3**^ **s**^**−1**^**)**
A	*T*_87_=35±3, *T*_85_=15±1, kick out ^85^Rb	*τ*_A_=74±12	(5.9±1.1) × 10^−11^	5.6 × 10^−11^	5.7 × 10^−11^
	*T*_87_=47±3, *T*_85_=27±2, kick out ^85^Rb	*τ*_A_=121±13	(6.5±0.8) × 10^−11^	5.9 × 10^−11^	
B	*T*_87_=35±3, *T*_85_=15±1, kick out ^85^Rb	*τ*_B_=70±8	(6.3±0.9) × 10^−11^	6.8 × 10^−11^	1.1 × 10^−10^
	*T*_87_=35±3, *T*_85_=15±1, kick out ^87^Rb	*τ*_B_=67±9	(6.6±1.0) × 10^−11^		
C	*T*_87_=35±3, *T*_85_=15±1, kick out ^85^Rb	*τ*_C_=1,800±250	(2.4±0.4) × 10^−12^	3.2 × 10^−12^	0.8 × 10^−12^
